# In-Depth Understanding of the Impact of Material Properties on the Performance of Jet Milling of Active Pharmaceutical Ingredients

**DOI:** 10.3390/pharmaceutics17091197

**Published:** 2025-09-15

**Authors:** Viktor Bultereys, Kensaku Matsunami, Laure Descamps, Roel Mertens, Alain Collas, Ashish Kumar

**Affiliations:** 1 Pharmaceutical Engineering Research Group (PharmaEng), Department of Pharmaceutical Analysis, Ghent University, Ottergemsesteenweg 460, 9000 Ghent, Belgium; viktor.bultereys@ugent.be (V.B.); laure.descamps@ugent.be (L.D.); 2 Janssen Pharmaceutica NV, a Johnson & Johnson Company, Turnhoutseweg 30, 2340 Beerse, Belgium; rmerten4@its.jnj.com (R.M.); acollas@its.jnj.com (A.C.)

**Keywords:** pharmaceutical process design, spiral air jet milling, material properties, statistical analysis, population balance model, active pharmaceutical ingredients

## Abstract

**Background/Objectives:** Among different milling techniques, spiral air jet milling can produce finer particles without the use of solvents or additives, thereby improving the bioavailability and content uniformity of the final dosage form. However, milling can complicate downstream processability of active pharmaceutical ingredients (APIs) due to reduced bulk powder flowability and post-milling lump formation. Process settings are often optimized only for particle size reduction, without sufficient consideration of manufacturability, largely because of limited API availability and a lack of knowledge about influential material properties. This study aimed to investigate the impact of material properties and process settings on milling performance and downstream manufacturability. **Methods:** Four APIs, examined in a total of eight grades, were characterized for their bulk mechanical properties and compression energy parameters using a compaction simulator. These grades were subjected to milling experiments within a design-of-experiments framework. Statistical analyses were performed, and population balance models (PBMs) were developed and calibrated for each experiment to link material properties and process settings to milling outcomes. **Results:** A higher gas flow rate was identified as the most significant contributor to particle size reduction. The influence of mechanical properties, particularly Young’s modulus and Poisson’s ratio, was evident and correlated with unmilled particle sizes. PBM analyses showed that a higher gas feed rate decreased the critical particle size for breakage, while intrinsic mechanical properties affected the breakage rate function. **Conclusions:** By integrating material properties and process settings into PBM analyses, specific breakage mechanisms could be identified. These findings provide a framework for optimizing jet milling not only for particle size reduction but also for downstream processability of APIs.

## 1. Introduction

Achieving a desired particle size distribution robustly is an important quality-related property during the development of active pharmaceutical ingredients (APIs) toward final drug product performance. When this is challenging to achieve during the crystallization and isolation steps of the API, a particle size reduction step is often introduced to normalize the API material attributes [1,2]. Milling, also referred to as grinding, comminution, or pulverization, is currently considered the standard unit operation for the size reduction of drug particles [3,4]. However, the complexity of the milling and subsequent classification operations is not yet fully understood. This leads to a predominantly trial-and-error approach in milling process design, which requires substantial amounts of material [5]. Given the high cost and limited availability of newly synthesized APIs during development, there is a demand for predictive models to alleviate the extensive experimental burden in process design. Yet, achieving this is challenging due to the complex nature of particle breakage, which is influenced by various intertwined factors such as material properties and process parameters [2].

Milling is a versatile process, offering various modes depending on the application. Typically, milling techniques are categorized into dry and wet milling. As the name suggests, dry milling is performed on dry powder, eliminating the need for a compatible solvent to disperse the particles [6]. Spiral jet milling is a dry milling method favored in the pharmaceutical industry because of its simplicity in design. The utilization of high-energy gas flows to induce particle-to-particle collisions eliminates the need for high-speed rotational mechanical components, which are prone to contamination with water, making them more difficult to clean and operate. The first patent was granted in the 1930s, and since then, this method has been widely employed in the micronization of pharmaceuticals. A spiral jet mill comprises a cylindrical milling chamber with a central outlet, which acts as a static classifier, and tangential nozzles, known as grinding nozzles, through which compressed gas is pushed [2]. Within the chamber, gas jets emitted from the grinding nozzles accelerate the particles, creating a vortex that results in particle breakage [4,7]. Inside the vortex, two counteracting principles act on the particles: centrifugal forces versus drag and lift forces. At a certain particle size, the drag and lift forces surpass the centrifugal force, causing the particle to exit the milling chamber through the central outlet [2].

Compared to impact mills, few models have been developed for jet mills due to the added complexity of fluid interactions [8]. Nonetheless, there is a need for jet mill models both to better understand the principles behind the process and to predict the final particle sizes. Efforts in modeling spiral jet mills have focused on various approaches, including specific energy correlations, force balances, discrete element method (DEM), population balance modeling (PBM), and computational fluid dynamics (CFD) [2]. Midoux et al. [9] found a correlation between the specific energy consumption and the specific surface area (SSA) of the particles in the form of a power function. This type of empirical modeling is easy to apply, but it does not consider the material properties [8]. Another approach is based on the force balance acting on the particles. By rearranging this balance, MacDonald et al. [10] derived an expression for the particle cut size as employed in the design of cyclonic separators, defined as the particle size at which particles leave the milling chamber through the central outlet. This equation is helpful in process modeling and scale-up and provides a means to improve energy efficiency. However, deriving multiple empirical constants requires complex analysis, which may not be feasible for all datasets [2,10].

PBM, a mesoscale method for tracking particle size distributions, has been applied to milling and to other particulate processes such as granulation, mixing, and dissolution [8]. It offers a balance between the mechanistic insights of DEM and the simplicity of empirical approaches [11]. Multiple efforts have been made to apply PBM to jet mills, including compartmental approaches consisting of comminution, central, and classifier zones [12], a steady-state PBM expressed in terms of mass fraction [13], and a PBM based on the Markov principle [14]. Despite numerous studies, there remains a missing link between PBM parameters representing breakage phenomena and raw material properties as well as process settings.

DEM provides detailed particle-level insights, often using bounded particle [15] or replacement methods [16], but it requires a high computational load [8,17,18]. Hybrid approaches, such as PBM-DEM and CFD-DEM, enable multi-scale modeling of breakage and fluid-particle interactions [2,5,17]. However, current mechanistic models still fall short of capturing the full impact of material properties on breakage [19].

This study aimed to identify correlations between API properties and milling performance and to apply this knowledge to a PBM to predict the spiral jet milling process.

## 2. Materials and Methods

### 2.1. Materials

During this study, four different APIs were used as model compounds, namely Domperidone, Ketoconazole, Metformin, and Indometacin, all provided by Johnson & Johnson Belgium (Beerse, Belgium). These APIs were selected based on their diverse physicochemical properties (salt forms, hydrophilicity/hydrophobicity, melting points), the availability of literature data for validation, and suitability for mechanistic investigations (e.g., known polymorphic forms and crystallization procedures). These compounds also represent the typical processability characteristics of new pharmaceutical products, while allowing sufficient material quantities for preparing the required morphologies and particle size distributions. Some of the APIs were recrystallised to different grades, characterized by different size distributions and habits. The complete list of materials used in the study is provided in Table 1.

Here, the term habit explicitly refers to the crystal particle morphology. For ketoconazole and domperidone, one original grade and one habit-modified grade were available, which already showed distinct differences in d-values. Indomethacin was only studied in a single grade. Metformin was examined in three different habits: elongated needle-like crystals (habit 1), more prismatic block-like crystals (habit 2), and flat plate-like crystals (habit 3).

### 2.2. Material Properties

For each API, mechanical properties (Young’s modulus and Poisson ratio) and energy parameters (elastic recovery and specific work of compaction) were determined. To assess these in-die properties, tablets with a solid fraction of one were prepared using the hydraulic compaction simulator HB 1088-C (Huxley Bertram Engineering, Cambridge, UK). Before compaction, external lubrication was performed with magnesium stearate to reduce friction as much as possible. The simulator used round, flat-faced punches with a diameter of 6 mm. Load cells and linear variable differential transformer sensors were integrated into the punches to accurately monitor the position of the load applied by both the upper and lower punches. The die was equipped with two die-wall sensors to measure the pressure exerted on the die-wall during the compaction process. These sensors were positioned at a 120-degree angle from each other and were placed at the same depth within the die. The average value obtained from both sensors was used to represent the radial pressure data. The compaction profile was determined by applying the Rippie–Danielson Equation [20].

The Poisson ratio (ν) and Young’s modulus (*E*) were derived from Equations (1) and (2), respectively:

(1) $$d\left(\sigma_{z}\right)=\frac{1-\nu }{\nu }d\left(\sigma_{r}\right)$$

(2) $$d\left(\sigma_{z}\right)=\frac{E(1-\nu )}{\left(1+\nu )(1-2\nu \right)}d\left(\epsilon_{z}\right)$$

where $\sigma_{z}$ is the axial stress, $\sigma_{r}$ the radial stress, and $\epsilon_{z}$ the axial strain, calculated based on the powder density before compression $\rho_{0}$ and the actual powder density during unloading ρ, as depicted in Equation (3).

(3) $$\epsilon_{z}=ln\left(\frac{\rho }{\rho_{0}}\right)$$

**Figure 1:**
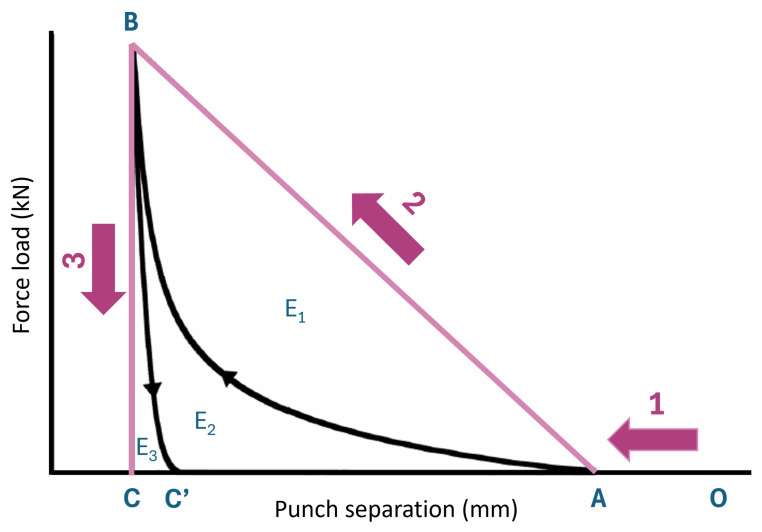
Typical force-displacement curve during tablet compression, consisting of (1) particle rearrangement, (2) compaction, and (3) decompression. The coloured triangle depicts an ideal process (Adapted from [23]).

The energy parameters of the APIs were established following the methodology proposed by [21], which relies on calculating the area under the force-displacement curve (Figure 1). As illustrated in the figure, the compression process can be divided into three distinct phases: (1) particle rearrangement, (2) compaction, and (3) decompression. However, the idealized force-displacement curve depicted in the colored triangle in the figure does not reflect reality. In practice, energy losses occur during both the compaction (E1) and decompression (E3) phases, which are attributed to friction and elastic recovery, respectively. By computing the area under the curve (AUC) for the individual phases, the energy associated with each phase was calculated, providing insights into the elastic and plastic characteristics of the material. In this study, AUCs were calculated using numpy.gradient in Python [22] from temporal data of punch separation and force loading during compaction and decompression. Table 2 presents an overview of the energy parameters calculated and how they were computed.

### 2.3. Milling Experiments

#### 2.3.1. Spiral Jet Mill

**Figure 2:**
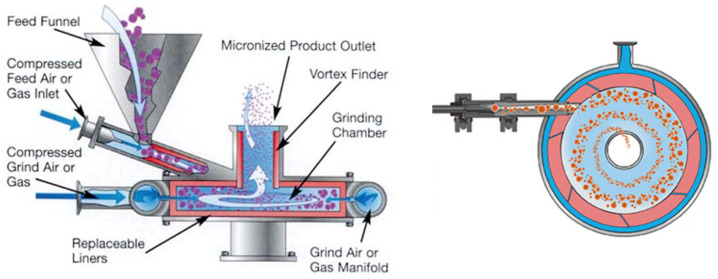
Overview of the spiral jet milling process from a side (**left**) and top (**right**) view of the milling chamber (Adapted from [24]).

The materials were ground using an Alpine^®^ spiral jet mill 50AS (Hosokawa, Augsburg, Germany). Figure 2 gives an overview of the spiral jet milling process from a side and top view. The diameter and height of the grinding chamber were 50 and 4.5 mm, respectively, with a standard blowing-out nozzle. There were four grinding nozzles, each with a diameter of 0.8 mm and a nozzle pitch of 50°. Nitrogen was used as the grinding gas. The injector pressure gas entered through a nozzle of 0.9 mm and was varied in the same way as the gas flow rate (GFR). The material was fed into the jet mill using a pitchFB ZD twin screw loss-in-weight feeder (ThreeTec, Seon, Switzerland) with double concave 4.5 mm pitch screws. Before feeding the material, it was passed through a 1 mm sieve to break up agglomerates. After the experiment, the (un)milled samples were stored in glass bottles.

For the spiral jet mill experiments, a $2^{2}$ full factorial design was constructed with GFR and mass flow rate (MFR) as the changing variables. The range of the GFR (2.2 to 9.0 m^3^/h) was determined based on the operating ranges of the equipment. The levels correspond to a gas pressure of 1 bar and 6.5 bar, respectively, and the centerpoint (CP) value of 5.6 m^3^/h corresponds to a pressure of 4 bar. The range of the MFR (5 to 15 g/min) was determined based on earlier feeder trials. The desired MFR could be converted into screw speed, which was checked and adjusted during the DoE. This was done to eliminate any potential day-to-day variation in the mass flow rate due to multiple factors, such as temperature, relative humidity, and tribo-electrification behavior.

#### 2.3.2. Particle Size Distribution (PSD) Measurements

Novel PSD characterization methods were developed in this study to measure fine particle sizes accurately and robustly. Laser diffraction measurements were conducted using a Mastersizer 3000 (Malvern Panalytical, Malvern, UK) equipped with a hydroMV wet sample module and operated at a stirrer speed of 2000 rpm. Samples were analyzed the day after jet milling experiments. For domperidone, ketoconazole, and indomethacin, a small amount of the material was wetted with Tween20 to distribute the powder. The procedure involved adding the samples drop by drop to a sample unit filled with water until the obscuration level ranged between 5 and 15%. For Metformin HCl, which is a water-soluble and hygroscopic material, the powder was mixed with 5 mL of a 0.1% lecithin (Sigma-Aldrich, Overijse, Belgium) in miglyol (IOI Oleochemical, Hamburg, Germany) solution and then sonicated for 5 min to break up any agglomerates. The resulting mixture was then added dropwise to a sample unit containing 0.01% lecithin/miglyol solution. The refractive index (RI) of the dispersant was set at 1.33 for water and 1.44 for miglyol. The RI and absorption index (AI) of the APIs are listed in Table 3. Finally, the PSD of the samples was determined, and the 10th (dv10), 50th (dv50), and 90th (dv90) percentiles of the cumulative volume distribution were computed. Three different samples were measured from each (un)milled fraction, with ten distinct measurements recorded for each sample. As indicators of the milling performance, the ratios of unmilled to milled dv10, dv50, and dv90 values were calculated.

### 2.4. Data Analysis

The results of the milling experiments were further analyzed by performing multiple linear regression (MLR) using the MODDE^®^ software 12.1 of Sartorius. For each API, MLR was conducted for three different outputs: dv10, dv50, and dv90. The significant factors were identified and contour plots were constructed.

In the next step, correlations between the material properties and the milling performance were sought. For this, the ratio of unmilled to milled dv-values was used as a parameter representing the milling performance. The material properties considered were the in-die properties: Young’s modulus, Poisson ratio, elastic recovery, and specific work of compaction. Using Python 3.11.5, multiple plots were generated to explore potential correlations between these material properties and milling performance. Additionally, a principal component analysis (PCA) and partial least squares (PLS) analysis were performed using the SIMCA^®^ software 16 of Sartorius. For the PCA, the material in-die properties were used as variables. The PLS was performed with the material in-die properties, initial dv-values, GFR, and MFR as variables, and the milling performance indicators as responses.

The performances of the developed data-driven models were judged based on goodness-of-fit $R^{2}$ and goodness-of-prediction $Q^{2}$, as described in Appendix A.2.

### 2.5. PBM-Based Analysis

A PBM was utilized to understand milling performance mechanistically and link it with mechanistic properties and process settings. All of the model framework developments and data analyses in this section were performed using the SciPy 1.13.1 package in Python 3.11.5 [25].

#### 2.5.1. Population Balance Equations

Based on the population balance equations described by [11], an identifiable PBM was developed by simplifying PBM kernels and experimentally identifying some parameters. The temporal change in the number of particles of size *x* at time *t*, N(x,t) [−], is described by Equation (4):

(4) $$\frac{dN(x,t)}{dt}={\dot{N}}_{\mathrm{in}}\left(x\right)-{\dot{N}}_{\mathrm{out}}\left(x,t\right)-S\left(x\right)N\left(x,t\right)+\int_{x}^{\infty }b(x,\varepsilon )S(\varepsilon )N(\varepsilon ,t)d\varepsilon$$

where ${\dot{N}}_{\mathrm{in}}$ [s^−1^] and ${\dot{N}}_{\mathrm{out}}$ [s^−1^] represent the number of particles of size *x* entering and exiting per unit time, respectively. The functions *S* [s^−1^] and *b* [−] denote the specific breakage rate and the breakage distribution function, which express the number of particles of size *x* broken from a particle of size ε. To solve the PBM in Equation (4), particle size was discretized into *n* grids, as shown in Equation (5):

(5) $$\frac{dN_{i}}{dt}={\dot{N}}_{i,\mathrm{in}}-{\dot{N}}_{i,\mathrm{out}}-S_{i}N_{i}+\sum_{j=i+1}^{n}b_{i,j}S_{j}M_{j}$$

with *i* and *j*, the size class indices run from class 1, which contains the smallest particles, to class *n*, which contains the biggest particles.

The inlet term can be defined by the PSD of the unmilled material, $f_{\mathrm{unmil}}$ [−], the actual powder density, ρ [kg m^−3^], and MFR, ${\dot{m}}_{s}$ [kg s^−1^], as shown in Equation (6):

(6) $${\dot{N}}_{i,\mathrm{in}}=\frac{f_{i,\mathrm{unmil}}\cdot {\dot{m}}_{s}}{\rho \left(\frac{4}{3}\pi x_{i}^{3}\right)}$$

A classification curve and the scale factor *k* [s^−1^] describe the outlet term due to its self-classifying nature. The cumulative log-normal distributions, consisting of $\mu_{c}$ and $\sigma_{c}$, were used for the curve based on the literature [11,12].

(7) $${\dot{N}}_{i,\mathrm{out}}=N_{i}\cdot \frac{k}{2}\left[1-\mathrm{erf}\left(\frac{\log\left(x_{i}\right)-\mu_{c}}{\sqrt{2}\sigma_{c}}\right)\right]$$

The breakage rate was described based on [26] with the presence of the critical breakage particle size, $x_{\mathrm{crit}}$ [μm], as presented in Equation (8):

(8) $$S_{i}=\left\{\begin{array}{cc} 0 & |x_{i}<x_{\mathrm{crit}}\\ \alpha {\left(\frac{x_{i}}{x_{n}}\right)}^{\lambda } & |x_{i}\geq x_{\mathrm{crit}} \end{array}\right.$$

where α [s^−1^] and λ [−] are the coefficients of the breakage rate. As follows, the breakage distribution function was simplified from the literature [11,12]:

(9) $$b_{i,j}=B_{i,j}-B_{i-1,j}\;\mathrm{with}\;b_{1,j}=B_{1,j}$$

(10) $$B_{i,j}={\left(\frac{x_{i}}{x_{j}}\right)}^{\gamma }$$

where B*_i,j_* is the cumulative breakage distribution function. The parameter γ [−] is the coefficient related to the breakage distribution function. The simplification of the breakage distribution function was made to reduce the number of model parameters to be estimated, under the assumption that the milled PSDs are predominantly monomodal.

#### 2.5.2. Parameter Identification and Estimation

Parameter identifiability is one of the most critical issues for appropriate parameter estimation. A large number of model parameters often causes collinearities among them, making robust parameter estimation impossible. Since the PBM presented in Section 2.5.1 originally included 7 model parameters, the following steps were taken to decrease the number of parameters for the estimations, where the details and justifications are presented in Appendix A.3.

1. Fixing *k* due to its collinearity with α
2. Experimental determination of the classification curve ($\mu_{c}$ and $\sigma_{c}$)
3. Experimental determination of the critical breakage size $x_{\mathrm{crit}}$

After the identification of *k*, $\mu_{c}$, $\sigma_{c}$, and $x_{\mathrm{crit}}$, the remaining model parameters, i.e., α, γ, and λ, were estimated in the model calibration. The objective function for the model calibration was the sum of the maximum mean discrepancy (MMD) of volume-based and number-based PSDs, $MMD_{\mathrm{sum}}$, where details are described in Appendix A.4. The model was calibrated using particle swarm optimization, a stochastic global optimization algorithm [27].

#### 2.5.3. Identifiability Analysis

The identifiability analysis was performed to confirm the reliability of the parameter estimation results. In this study, the concept of [11,28] was used for the identifiability analysis, where the impact of varying the value of one parameter on the objective function was assessed. In the case of α, the function $IA(\alpha_{i})$ is calculated as follows:

(11) $$\begin{array}{ccc} IA(\alpha_{i})= & \min_{\gamma ,\lambda } & MMD_{\mathrm{sum}}\\ & s.t. & \alpha =\alpha_{i} \end{array}$$

The parameter identifiability of α was judged by plotting $IA(\alpha_{i})$. Specifically, IA should decrease monotonically as $\alpha_{i}$ approaches the estimated value in the model calibration. Beyond the estimated value, IA should increase monotonically. The model parameter was considered identifiable if the U-shaped behavior was observed.

#### 2.5.4. Linking PBM with Input Parameters

The identified and estimated PBM parameters were further analyzed to investigate the effect of material properties and process settings on milling performance. A PLS regression model was developed, where the model’s inputs were material properties and process settings, and the model outputs were the PBM parameters. The loadings of the PLS were utilized for the interpretation of the results.

## 3. Results and Discussion

### 3.1. Correlating Process Parameters and Milling Performance

**Figure 3:**
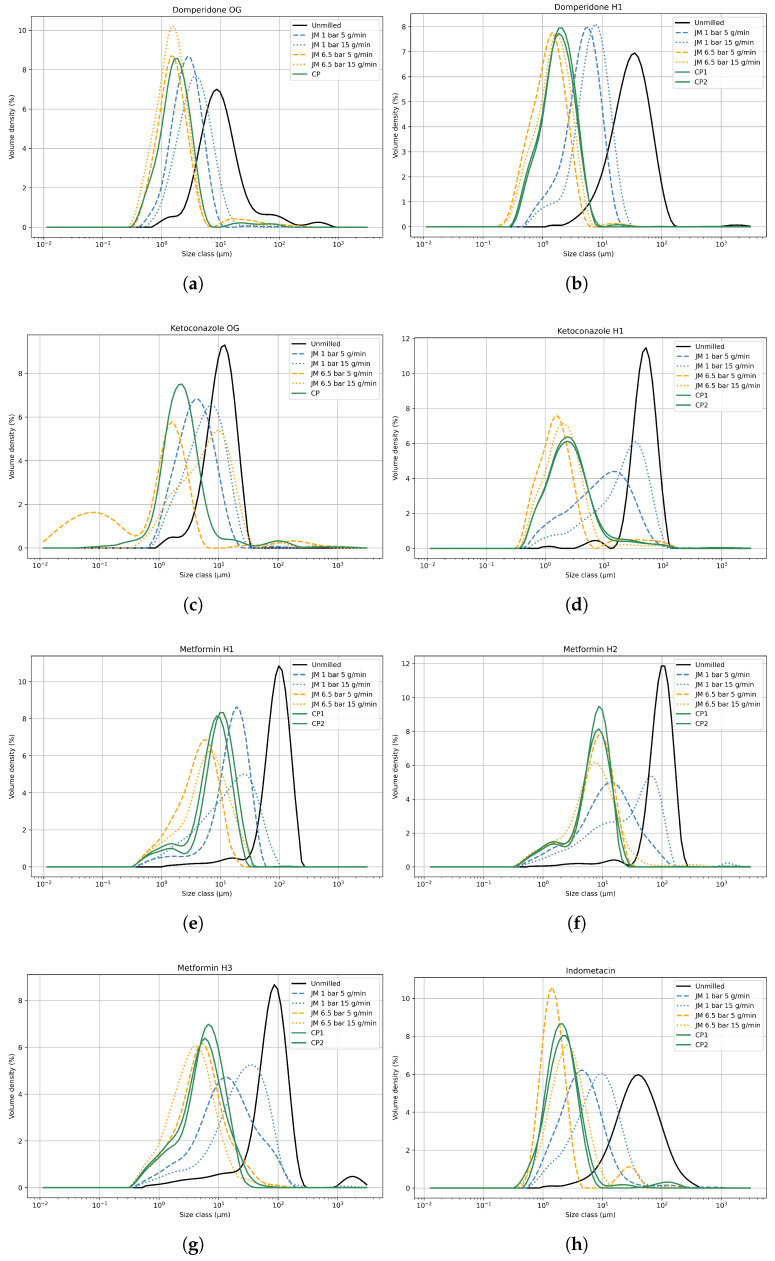
Particle size distributions of the jet mill experiments of (**a**) Domperidone OG, (**b**) Domperidone H1, (**c**) Ketoconazole OG, (**d**) Ketoconazole H1, (**e**) Metformin H1, (**f**) Metformin H2, (**g**) Metformin H3, and (**h**) Indometacin. For each API, the unmilled fraction and the fractions milled at different process settings are shown. CP represents the center point experiment.

Figure 3 displays the particle size distribution of both the unmilled powder and the fractions milled at various process settings for each API. The PSDs of the replicate centerpoint measurements closely resembled each other, indicating good reproducibility of the experiments and laser diffraction measurements. This is supported by the relative standard deviation (RSD) between the particle size distributions of the two replicates (Appendix A.1). All calculated RSD values were below 10%, indicating good agreement between the replicates, with the exception of Metformin H1 and dv90 of Ketoconazole H1.

The higher RSD for Metformin H1 is likely due to its tendency to form lumps, which complicates powder dispersion and introduces variability in laser diffraction measurements. Notably, such lump formation was not observed for Metformin H2 or OG, which may be due to improved powder handling during those measurements or differences in the degree of agglomeration that allowed more effective dispersion. In the case of Ketoconazole H1, its high cohesiveness and microfine particle size likely contributed to the elevated dv90 RSD, possibly due to insufficient de-agglomeration during measurement.

Additionally, in this study, unfiltered PSD data were used to preserve all measurement artifacts. This choice may have amplified the apparent variability for these challenging powders. In general, the PSDs had monomodal distributions, with some exceptions. In particular, Ketoconazole OG, which showed the lowest Young’s modulus, with 6.5 bar and 5 g/min, showed a bimodal PSD with a second peak at a very small particle size. This was potentially due to the formation of a large number of fines from particle attrition, a phenomenon observed with fragile materials. These fines can contribute to high powder cohesion and poor flow properties, posing challenges in downstream processing [1,6]. Similar observations were made for the Metformin habits, but to a lesser degree.

Left-skewing of the PSDs occurred for the Ketoconazole habits and for Metformin H1 and H2. This phenomenon could be attributed to particle attrition or post-milling particle agglomeration. It is also plausible that not all particles were uniformly reduced under these processing conditions, despite the classifier mechanism theoretically retaining the larger particles within the milling chamber. This highlights a limitation in the self-classifying nature of the spiral jet mill. At higher MFR and lower GFR, larger particles could potentially be pushed out through the classifier outlet due to choking of the milling chamber [12,29].

Table 4 provides an overview of the dv10, dv50, and dv90 response models for each API obtained by MLR, along with their corresponding R^2^ and Q^2^ values. The response distributions of Ketoconazole H1 (dv10, dv50), Metformin H2 (dv50, dv90), Metformin H3 (dv10, dv50), and Indometacin (dv50) exhibited skewness, prompting the application of logarithmic transformation. All the models exhibited acceptable R^2^ values (≥0.6), suggesting a good fit to the data. However, half of the models displayed low Q^2^ values (≤0.5) with a difference larger than 0.3 compared to R^2^, indicating poor predictive ability [30]. Consequently, the response models could not be used as predictive models. To use them for prediction, MLR needs to be replaced by advanced machine learning techniques, e.g., random forest or Gaussian process regression.

**Figure 4:**
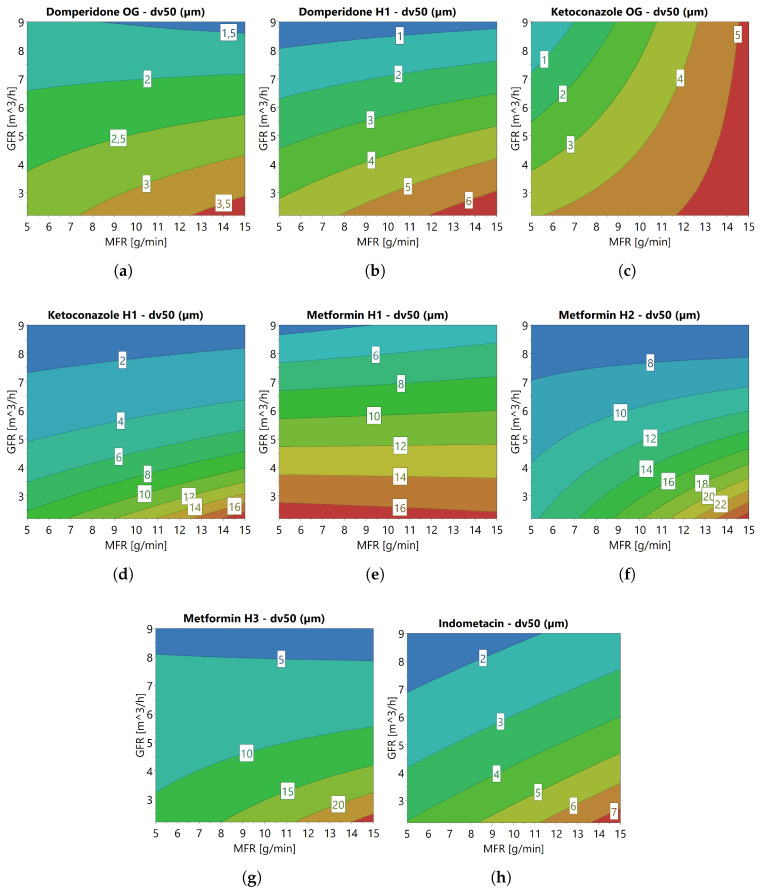
Contour plots of the dv50 response after jet milling of (**a**) Domperidone OG, (**b**) Domperidone H1, (**c**) Ketoconazole OG, (**d**) Ketoconazole H1, (**e**) Metformin H1, (**f**) Metformin H2, (**g**) Metformin H3, and (**h**) Indometacin. The contour plots were constructed based on the model, including both GFR and MFR.

The models instead served as indicators of the influence of GFR and MFR on milling performance. The models confirmed that higher GFR and lower MFR generally result in smaller final particle sizes. While [9] suggests the existence of an optimal MFR, the data obtained in this study did not support this claim. In the response models for most APIs, including the Domperidone habits, Ketoconazole H1, and the Metformin habit grades, GFR emerged as a significant factor, whereas MFR did not, with the dv90 response for Metformin H1 being an exception. Observations from contour plots (Figure 4) and PSDs (Figure 3) for these APIs indicated that the influence of MFR on final particle size diminished with increasing GFR. Conversely, for Indometacin, both GFR and MFR were significant in the response models, as evidenced by the contour plots and PSDs showing a noticeable effect of MFR even at higher GFR. The results obtained for Ketoconazole OG were substantially different from those of the other APIs, with MFR significantly impacting the final particle size, while GFR did not, except in the dv10 response. This exception could be due to the presence of a large number of fines generated during one of the experiments, which significantly influenced the dv10 value and may explain the deviating dv10 model. There was no definitive explanation for the divergent results for Indometacin and Ketoconazole OG compared to the other APIs. It is plausible that this disparity could be due to distinct material behavior not yet captured or experimental errors not accounted for.

### 3.2. Correlating API Properties and Milling Performance

**Figure 5:**
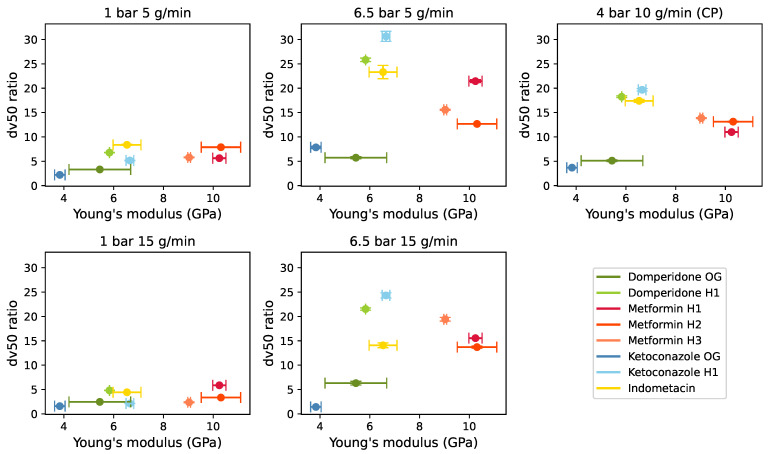
Correlation plot of the dv50 milling performance parameter in the function of Young’s modulus.

**Figure 6:**
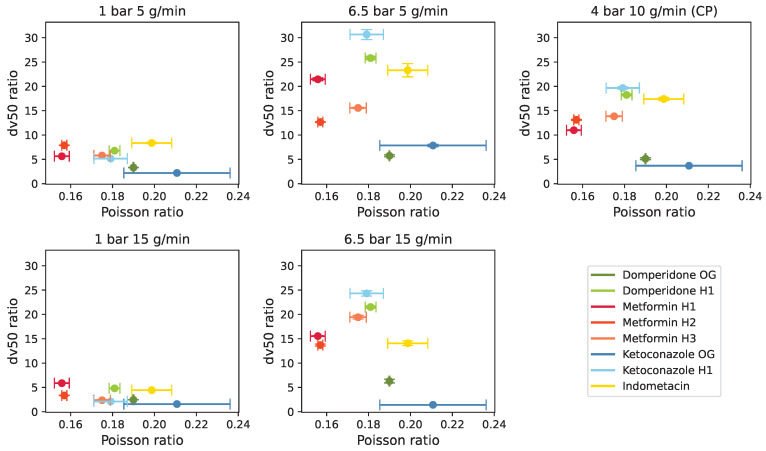
Correlation plot of the dv50 milling performance parameter in the function of Poisson ratio.

Correlation plots were constructed to investigate relationships between the in-die material properties and milling performance, depicted as the ratio of unmilled to milled dv50 values. Figure 5 and Figure 6 revealed subtle trends between milling performance and material properties, suggesting higher milling performance at higher Young’s modulus and lower Poisson ratio, aligning with theoretical expectations [31]. Nonetheless, these trends are not definitive and should be interpreted with caution.

**Figure 7:**
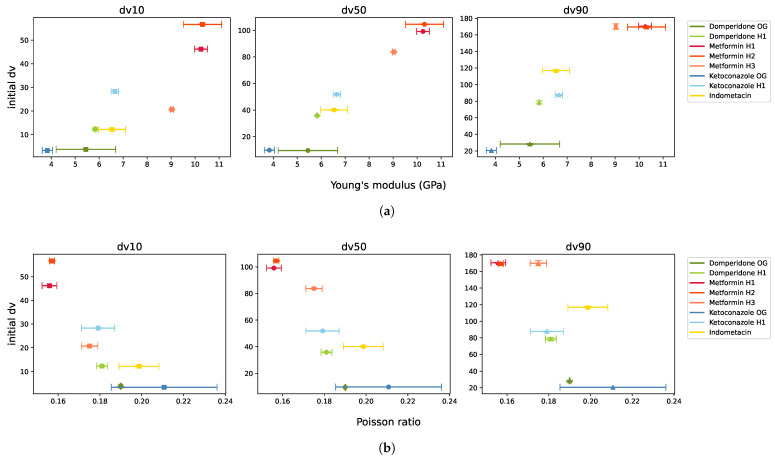
Initial particle size in the function of (**a**) Young’s modulus and (**b**) Poisson ratio, illustrating their relationship.

Young’s modulus and Poisson ratio seemed to be confounded with the initial particle size, as depicted in Figure 7. Young’s modulus was roughly proportional to particle size, while Poisson ratio was inversely proportional, consistent with the literature findings [32,33]. The obtained PSDs were measured from the particles exiting the mill, with the classifier ensuring that only particles below a certain size left the milling chamber. Consequently, larger initial particles were reduced more in size, resulting in a higher ratio of unmilled to milled dv values.

Hence, the purportedly higher milling performance associated with higher Young’s modulus or lower Poisson ratio may be attributed to initial particle size rather than intrinsic material properties. As material properties were solely determined at the initial particle size, it was feasible to explore the link between particle size and material properties across different APIs. To experimentally validate this link, material properties should be measured at multiple particle sizes for a single API. For example, Ketoconazole measured at two size grades (microfine vs. coarser H1) showed an increase in Young’s modulus from 3.83 GPa to 6.65 GPa at a solid fraction of one, corresponding to an increase of approximately 74%, despite both being the same polymorph.

**Figure A2:**
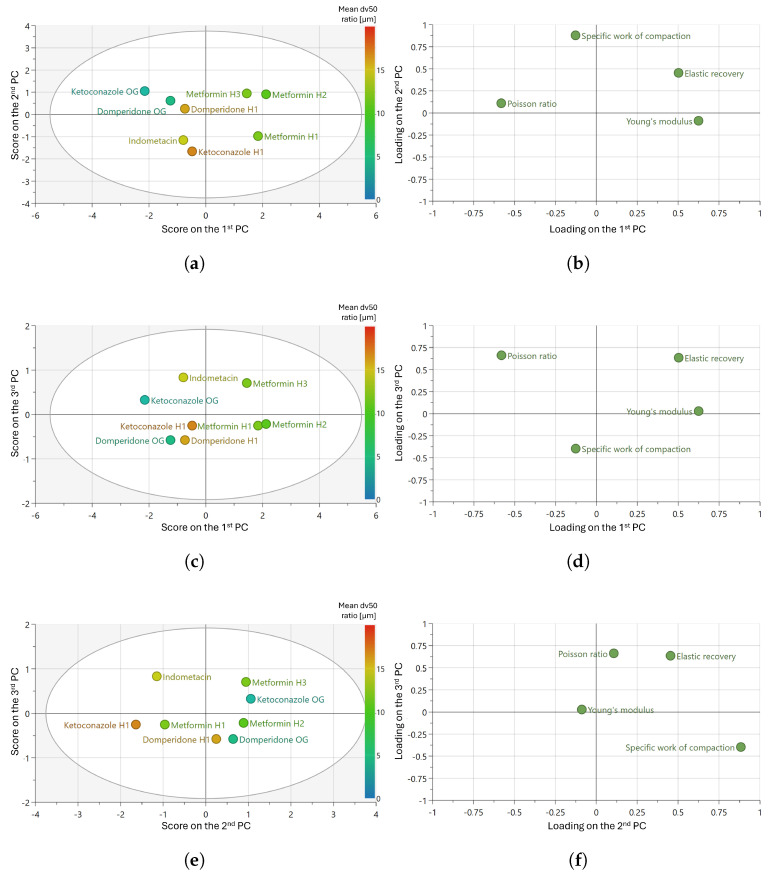
(**a**–**f**) PCA score and corresponding loading plots. Each row represents a different combination of principal components (PCs; 2-1, 3-1, and 3-2). APIs are colored according to their mean ratio of unmilled to milled dv50 values.

PCA was performed to confirm the observed trends in the correlation plots. Score plots were color-coded according to milling performance (Figure A2, Appendix A), excluding the outlier sample of Ketoconazole OG (6.5 bar–5 g/min) discussed earlier. Based on the model fit summary (Table 5), three principal components (PCs) were selected. Although Q^2^ continued to increase with a fourth component, the three-component model achieved Q^2^ > 0.9, close to R^2^, indicating excellent predictivity [34].

The score plots did not reveal a clear separation between better- and worse-performing APIs, yet a gradual decline in milling performance was evident along the second principal component. Comparison with the corresponding loading plot suggested a negative correlation between the specific work of compaction and milling performance, though this relationship should be interpreted with caution. Furthermore, the first principal component distinctly separated the Metformin habits from the other APIs, indicating higher Young’s modulus and elastic recovery, as well as lower Poisson ratio.

PLS analysis was conducted to further investigate the impacts of in-die properties, initial particle size, and process settings on milling performance. As in PCA, the outlier sample of Ketoconazole OG was excluded from the analysis. Two principal components were selected because adding a third decreased Q^2^ and widened the gap between R^2^ and Q^2^ (Table 5). With two components, R^2^ and Q^2^ values exceeded 0.5, indicating moderate predictivity [35]. The distance-to-model plot (Figure A4, Appendix A.5) flagged two Metformin H3 samples milled at 6.5 bar as borderline outliers, though no clear explanation was found.

**Figure 8:**
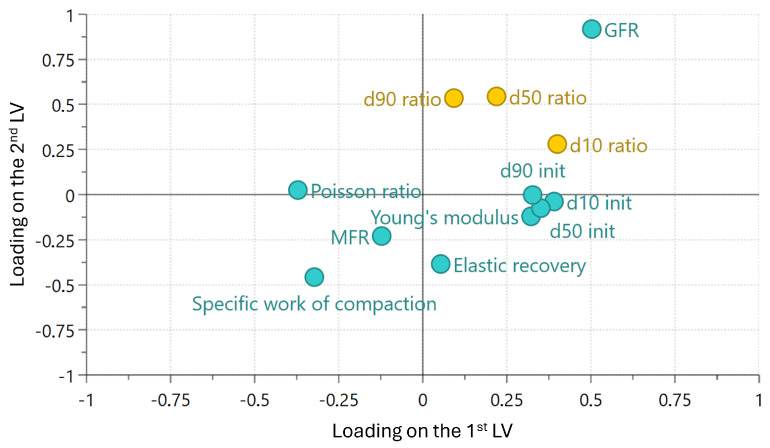
PLS loading plot at the first and second latent variables (LVs). Blue and yellow circles represent input and output parameters of PLS, respectively.

**Figure A3:**
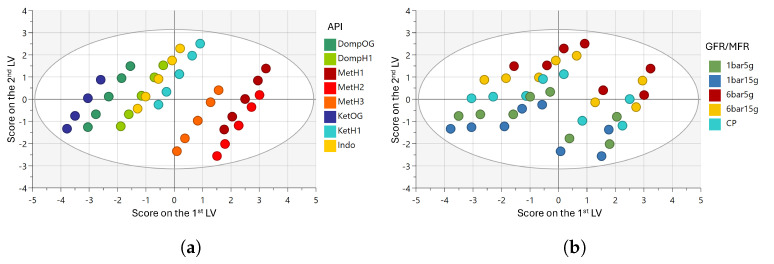
PLS score plots: (**a**) colored by API and (**b**) colored by milling process settings.

**Figure A4:**
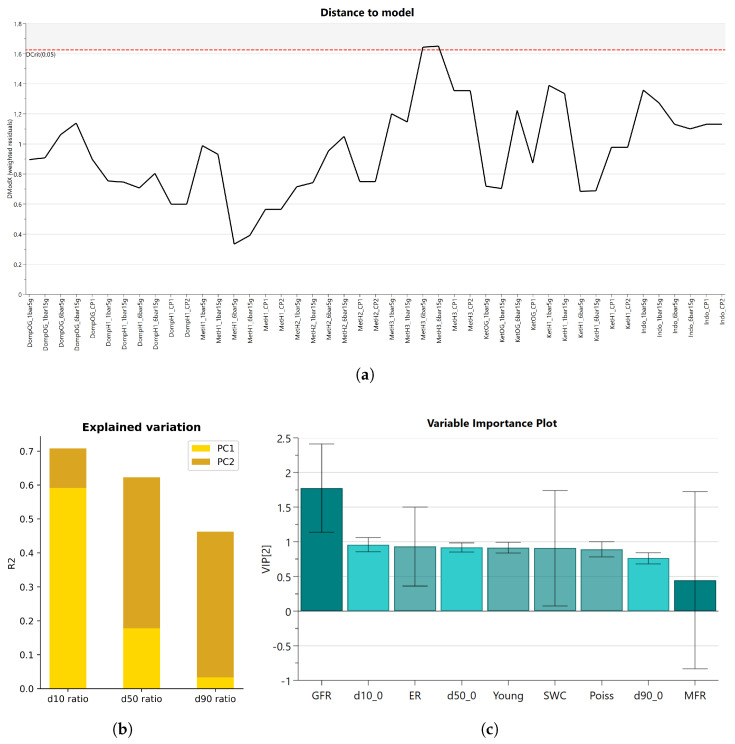
Supplementary PLS plots: (**a**) distance to model plot, (**b**) explained variation plot, and (**c**) variable importance plot. d10_0, d50_0 and d90_0 refer to the initial dv values. ER, Young, SWC and Poiss refer respectively to the elastic recovery, Young’s modulus, specific work of compaction and Poisson ratio.

The score plots (Figure A3, Appendix A.5) revealed that PC1 was primarily driven by material properties, while PC2 was dominated by process settings. Consistent with the MLR results, the loading plot (Figure 8) indicated that GFR had a stronger influence than MFR, which was further reinforced by the variable importance plot (Figure A4, Appendix A.5). Furthermore, the loading plot also confirmed the confounding of initial particle size with Young’s modulus and, more indirectly, with Poisson ratio.

Similar to the PCA results, the loading plot (Figure 8) suggested that the specific work of compaction was a key variable negatively correlated with milling performance, though this impact has high uncertainty according to the variable importance plot (Figure A4, Appendix A.5). The importance of the in-die material properties and initial particle size increased when smaller particle size distribution percentiles were considered. This was confirmed by how much each principal component contributed to the explained variation of the responses, depicted in Figure A4 in the Appendix A.5. The dv90 ratio was primarily influenced by process settings (PC2), whereas the dv10 ratio was dominated by material properties (PC1). One possible explanation for this phenomenon is that process settings predominantly influence factors such as energy input and collision frequency, thereby mainly affecting larger particles, whereas material properties have more influence on processes like attrition or agglomeration, predominantly impacting fines and smaller particles.

### 3.3. PBM-Based Analysis

#### 3.3.1. PBM Calibration and Identifiability Analysis

**Figure 9:**
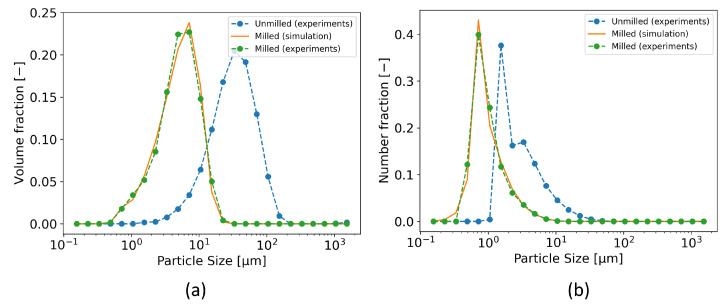
Calibration results of PBM for Domperidone H1 at 2.2 m^3^/h of GFR and 5 g/min of MFR: (**a**) volume-based PSDs and (**b**) number-based PSDs.

PBM successfully captured both volume-based and number-based PSDs. All of the calibration results are summarized in Table A2 in the Appendix A.4. Calibration was successful for most cases as the majority of the milled products exhibited monomodal PSDs (Figure 3). As an example, Figure 9 shows the PBM calibration results for Domperidone H1 at 2.2 m^3^/h of GFR and 5 g/min of MFR. Some experiments that yielded high objective function values, e.g., Metformin H1 at 2.2 m^3^/h of GFR and 15 g/min of MFR, corresponded to products with broader distributions (see Figure 3). The lower calibration performance in these cases can be attributed to the simplification of the breakage distribution function. While the original function in [11,12] can capture bimodal distributions more accurately, its larger number of parameters decreased identifiability. A more flexible breakage distribution function may therefore be required to achieve accurate simulation across different APIs and process conditions.

**Figure 10:**
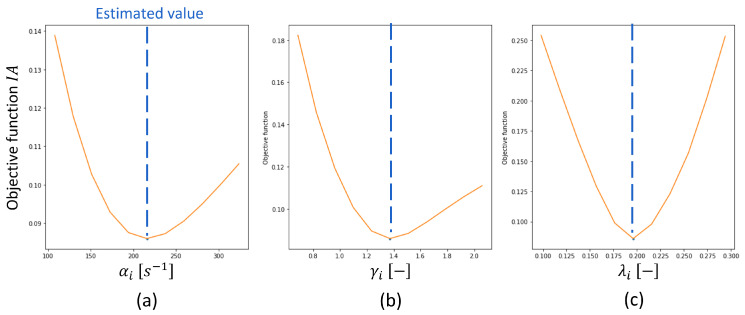
Results of the identifiability analysis of PBM for Domperidone H1 at 2.2 m^3^/h of GFR and 5 g/min of MFR: (**a**) α and (**b**) γ, and (**c**) λ.

The results of the identifiability analysis are presented in Figure 10, using the experimental data and the calibrated model for Domperidone H1 at 2.2 m^3^/h of GFR and 5 g/min of MFR. As described in Section 2.5.3, the objective function IA reached its minimum when the values were equivalent to the calibrated values. This result demonstrates that the proposed model and calibration procedure performed well in identifying all PBM parameters.

#### 3.3.2. PLS Analysis of PBM Parameters

**Figure 11:**
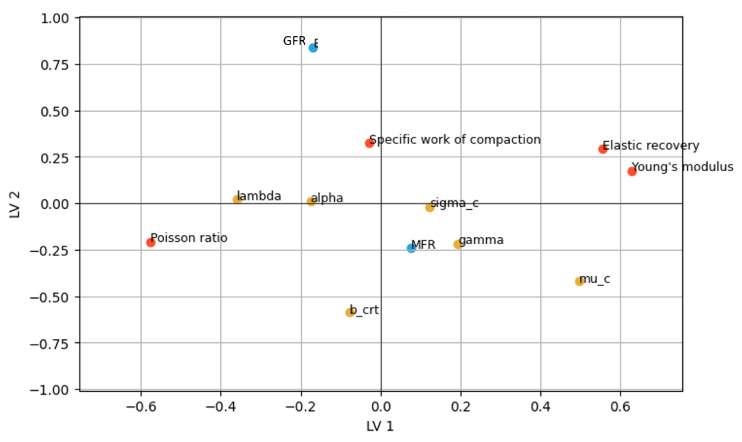
Loading plot of the PLS model analyzing the impact of material properties and process settings on PBM parameters. Orange, blue, and yellow dots represent material properties, process settings, and PBM parameters, respectively.

The loading plot of the PLS model is presented in Figure 11, where the impact of material properties and process settings on PBM parameters is visualized. The impact of process settings was captured by the second LV, whereas the first LV was mainly explained by mechanical properties, i.e., Young’s modulus and Poisson ratio. The energy parameters, i.e., elastic recovery and specific work of compaction, less to the first LV compared with mechanical properties, and less to the second LV compared with process settings.

For the PBM parameters, the critical breakage size $x_{\mathrm{crit}}$ was predominantly determined by the second LV, which could be linked with GFR. A strong negative correlation was observed between GFR and $x_{\mathrm{crit}}$; higher GFR reduced the critical particle size required for breakage, likely due to the increased kinetic energy available for generating new surface area. In addition to $x_{\mathrm{crit}}$, the breakage rate-related parameters, i.e., α and λ, were influenced only by the first LV. While process settings affected only the critical breakage size, the breakage rate of larger particles was entirely dependent on the material properties of the APIs.

Although the purpose of this section is similar to that of Section 3.2, hybrid modeling of PLS and PBM enabled an in-depth understanding of milling performance. The use of the ratio of unmilled to milled dv-values had two main limitations: (i) the effect of the unmilled PSDs on the indicator, and (ii) the representativeness of dv-values for the entire PSDs. In contrast, the PBM parameters were less dependent on the unmilled PSDs and were able to capture effects across the entire PSD. Furthermore, milling performance could be categorized into three factors: breakage rate over the size, breakage distribution functions, and classifying functions as a virtual screen. This categorization made it possible to understand how mechanical properties, energy parameters, and process settings influenced specific aspects of milling performance.

## 4. Conclusions

Toward the incorporation of manufacturability into the drug design process, this study investigated the relationship between the mechanical properties of APIs and their performance in the spiral jet milling process. The impact of material properties and process settings on milling performance was analyzed using a Design-of-Experiments approach, followed by statistical analysis, e.g., MLR and PLS, and PBM-based analysis, which combined mechanistic and data-driven approaches. The gas flow rate had a greater impact on milling performance, especially on the critical breakage size, than the mass feed rate. While statistical analysis faced challenges in isolating the contribution of mechanical properties due to their correlation with initial particle sizes, the PBM-based analysis revealed that they affected the breakage rate function.

The presented approach is valuable for designing milling processes for new APIs, as it considers not only process settings but also input material properties such as particle size and mechanical properties. In addition to statistical analyses, an identifiable PBM was developed to analyze the impact on milling performance. The integration of different analyses strengthened common findings, e.g., high GFR effect, and clarified some pitfalls, e.g., the need to account for initial PSDs in the analyses. These approaches demonstrated the feasibility of a more nuanced and systematic design of milling as well as process control by integrating them with process analytical technology (PAT) tools. For example, in relation to quality control, the results indicated that the Dv90 can be controlled by GFR through $x_{crit}$ reduction, while excessive fines (overmilling) could potentially be minimized by optimizing unmilled material properties, thus reducing unfavorable downstream complications.

Future studies should incorporate advanced measurement techniques that enable the determination of material properties across different particle sizes within the same API. This would help disentangle particle size effects from inherent material differences and lead to more robust conclusions about their impact on milling performance. In addition, dynamic information, e.g., the time-dependent outlet of milled APIs and milled PSDs during the start-up, as well as PSDs remaining in the mill, will enable more detailed and robust PBM development and PBM validation. Once the developed models achieve sufficient descriptive power, they may be applied to process optimization with fewer experiments [36] and can be linked with formulation performance, e.g., dissolution.

## Figures and Tables

**Table 1:** List of used materials in the study.

API	Form	Grade *	Abbreviation	dv10 [μm]	dv50 [μm]	dv90 [μm]
RS-Ketoconazole	I	H1	KetH1	28	52	88
RS-Ketoconazole	I	OG	KetOG	3	10	21
Metformin HCl	A	H1	MetH1	46	99	171
Metformin HCl	A	H2	MetH2	57	105	170
Metformin HCl	A	H3	MetH3	21	84	170
Domperidone	1	H1	DompH1	12	36	79
Domperidone	1	OG	DompOG	4	10	28
Indomethacin	gamma	OG	IndOG	12	40	117

* ‘H’ and ‘OG’ represent habit and original grade.

**Table 2:** Overview of the calculated energy parameters and how they were determined.

Parameter	Symbol	Unit	AUC	Equation
Work of elastic recovery	$W_{\mathrm{elastic}}$	J	BCC’ (E3)	
Work of compaction	$W_{\mathrm{compact}}$	J	ABC’ (E2)	
Work of compression	$W_{\mathrm{compress}}$	J	ABC (E3 + E2)	
Elastic recovery	ER	%		$\frac{W_{\mathrm{elastic}}}{W_{\mathrm{compress}}}\times 100$
Specific work of compaction	SWC	J/g		$\frac{W_{\mathrm{compact}}}{m_{\mathrm{tablet}}}$

**Table 3:** Refractive index (RI) and absorption index (AI) values of the different APIs.

API	RI	AI
Domperidone	1.64	0.02
Ketoconazole	1.64	0.05
Metformin HCl	1.58	0.10
Indometacin	1.68	0.01

**Table 4:** Response models based on the experimental jet mill data and their respective R^2^ and Q^2^ values. The underlined models indicate the logarithmic transformation of the response data.

	Model	R^2^	Q^2^
Domperidone OG	dv10 = 1.01 − 0.43 × GFR	0.844	0.550
	dv50 = 2.37 − 0.90 × GFR	0.799	0.430
	dv90 = 5.18 − 1.80 × GFR	0.668	0.024
Domperidone H1	dv10 = 0.80 − 0.82 × GFR + 0.64 × GFR^2^	0.892	0.569
	dv50 = 2.03 − 2.36 × GFR + 1.98 × GFR^2^	0.923	0.692
	dv90 = 4.50 − 4.84 × GFR + 4.06 × GFR^2^	0.922	0.687
Ketoconazole OG	dv10 = 1.15 + 0.40 × MFR − 0.69 × GFR + 0.26 × MFR × GFR	1.000	0.998
	dv50 = 3.70 + 1.68 × MFR	0.661	0.057
	dv50 = 9.83 + 4.60 × MFR	0.680	0.061
Ketoconazole H1	dv10 = 0.08 − 0.26 × GFR	0.741	0.304
	dv50 = 0.43 − 0.45 × GFR + 0.31 × GFR^2^	0.925	0.701
	dv90 = 9.28 − 21.07 × GFR + 18.31 × GFR^2^	0.879	0.515
Metformin H1	dv10 = 2.45 − 1.39 × GFR	0.701	0.163
	dv50 = 10.53 − 6.14 × GFR	0.944	0.882
	dv90 = 17.52 + 4.02 × MFR − 13.06 × GFR + 8.54 × GFR^2^	0.980	0.766
Metformin H2	dv10 = 1.76 − 0.64 × GFR + 0.59 × GFR^2^	0.809	0.237
	dv50 = 1.03 − 0.22 × GFR	0.600	0.037
	dv90 = 1.18 − 0.24 × GFR + 0.41 × GFR^2^	0.897	0.588
Metformin H3	dv10 = 0.17 − 0.23 × GFR + 0.19 × GFR^2^	0.860	0.441
	dv50 = 0.79 − 0.33 × GFR + 0.23 × GFR^2^	0.867	0.469
	dv90 = 16.71 − 32.17 × GFR + 37.27 × GFR^2^	0.835	0.340
Indometacin	dv10 = 1.05 + 0.30 × MFR − 0.52 × GFR + 0.39 × GFR^2^	0.993	0.897
	dv50 = 0.36 + 0.12 × MFR − 0.24 × GFR + 0.22 × GFR^2^	0.998	0.962
	dv90 = 4.94 + 3.53 × MFR − 6.38 × GFR + 7.14 × GFR^2^	0.955	0.278

**Table 5:** PCA and PLS summary of fit, showing the (cumulative) R^2^ and (cumulative) Q^2^ values with increasing number of principal components.

	PC	R^2^	R^2^ (cum)	Q^2^	Q^2^ (cum)
*PCA*	1	0.627	0.627	0.169	0.169
	2	0.293	0.920	0.160	0.302
	3	0.076	0.996	0.901	0.931
	4	0.003	0.999	0.955	0.997
*PLS*	1	0.267	0.267	0.234	0.234
	2	0.330	0.597	0.377	0.523
	3	0.048	0.645	−0.044	0.502

**Figure A1:**
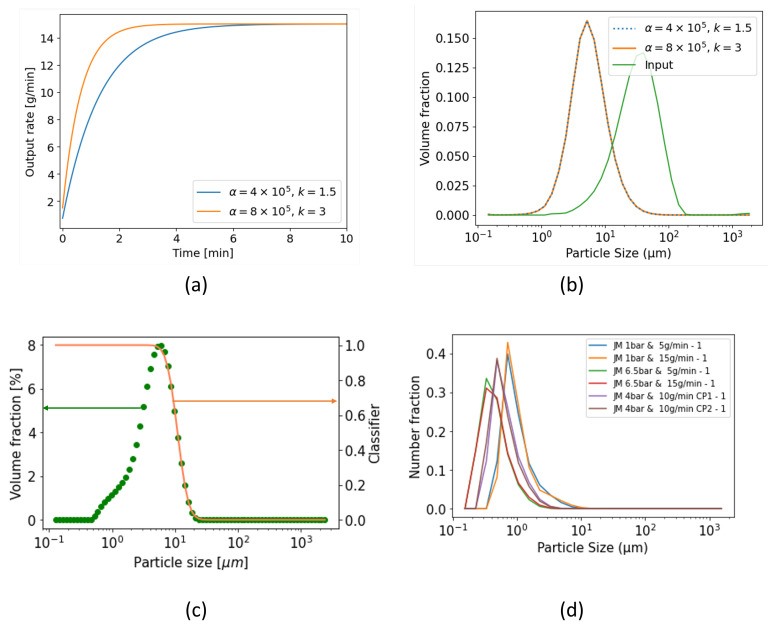
PBM development performed to improve the model identifiability: (**a**) temporal change of the output rate of milled particles with a fixed ratio of α and *k*, (**b**) PSDs of the milled products at the steady state with a fixed ratio of α and *k*, (**c**) the relation between the milled PSD and the classification curve, and (**d**) number-based PSDs of milled APIs.

## Data Availability

The data presented in this study are available on request from the corresponding author. The data are not publicly available due to confidential information.

## Abbreviations

The following abbreviations are used in this manuscript:

AI	Absorption index
API	Active pharmaceutical ingredient
AUC	Area under the curve
CFD	Computational fluid dynamics
dvXX	XXth percentile of the cumulative volume distribution
CP	Centerpoint
DEM	Discrete element method
GFR	Gas flow rate
LV	Latent variable
MMD	Maximum mean discrepancy
MLR	Multiple linear regression
MFR	Mass flow rate
PAT	Process analytical technology
PBM	Population balance model
PCA	Principal component analysis
PLS	Partial least squares
PSD	Particle size distribution
$Q^{2}$	Goodness-of-prediction
$R^{2}$	Goodness-of-fit
RI	Refractive index
RSD	Relative standard deviation
SSA	Specific surface area

## Appendix A

### Appendix A.1. Relative Standard Deviation of Centerpoint Experiments

**Table A1:** Relative standard deviation (RSD) between dv10, rdv50 and dv90 values of the two centerpoint particle size distributions of each material, expressed in %. Domperidone OG and Ketoconazole OG are not included in this table as only 1 centerpoint experiment was successful.

API	%RSD		
	**dv10**	**dv50**	**dv90**
Domperidone H1	6.17	3.73	1.13
Ketoconazole H1	0.19	2.67	18.63
Metformin H1	22.79	18.82	18.35
Metformin H2	9.82	3.52	8.80
Metformin H3	8.73	9.93	9.62
Indometacin	8.23	4.17	7.45

### Appendix A.2. Equations to Judge the Performance of the Data-Driven Models

(A1) $$R^{2}=1-\frac{\sum_{i=1}^{n}{\left(y_{i}-{\hat{y}}_{i}\right)}^{2}}{\sum_{i=1}^{n}{\left(y_{i}-\bar{y}\right)}^{2}}$$

(A2) $$Q^{2}=1-\frac{PRESS}{\sum_{i=1}^{n}{\left(y_{i}-\bar{y}\right)}^{2}}$$

where the parameters $y_{i}$, ${\hat{y}}_{i}$, and $\bar{y_{i}}$ represent the experimental and fitted values of *i*-th data, and the mean of all experimental data, respectively. The value of PRESS can be calculated by Equation (A3) for MLR and Equation (A4) for PLS:

(A3) $$PRESS=\sum_{i=1}^{n}{\left(\frac{y_{i}-{\hat{y}}_{i}}{1-X{\left(X^{⊤}X\right)}^{-1}X^{⊤}}\right)}^{2}$$

(A4) $$PRESS=\sum_{i=1}^{n}{\left(y_{i}-y_{i}^{\hat{c}v}\right)}^{2}$$

where X and $y_{i}^{\hat{c}v}$ are the matrix of the input variables and simulated values in cross-validation, respectively.

### Appendix A.3. The Strategy to Decrease the Number of PBM Parameters for the Estimations

1. Fixing *k* due to its collinearity with α
  The developed PBM was performed by two different sets of α and *k* with a fixed ratio, i.e., $\alpha =4\times 10^{5}$, k=1.5, and $\alpha =8\times 10^{5}$, k=3, as shown in Figure A1a,b. While the two simulations showed different start-up behavior (Figure A1a), the PSDs at the steady state were exactly overlapping (Figure A1b). Since only PSDs at the steady state were characterized in this study, *k* was fixed as 60 ${\dot{m}}_{s}$.
2. Experimental determination of the classification curve
  Figure A1b also suggests that large particles remained in the mill until they were broken into small particles. The assumption was made that the dominant phenomena affecting milled particles vary depending on their size relative to the peak of the PSD. For particles larger than the peak size, the classification curve of the outlet term predominantly influences their behavior. Hence, the classification parameters $\mu_{c}$ and $\sigma_{c}$ were estimated based on the PSDs of the particles larger than the peak size, as shown in Figure A1c.
3. Experimental determination of the critical breakage size $x_{\mathrm{crit}}$
  The physical meaning of the critical breakage size is the maximum size of the particles, which are never broken further. Hence, the number of particles smaller than $x_{\mathrm{crit}}$ is expected to be significantly smaller. As presented in Figure A1d, the number-based particle size distributions exhibited sharp peaks, which could be explained by $x_{\mathrm{crit}}$. The peak size of the number-based particle size distributions was used as $x_{\mathrm{crit}}$.

### Appendix A.4. The Objective Function for the Model Calibration and Calibration Summary

The objective function for the model calibration was the maximum mean discrepancy (MMD) between distributions *u* and *v*, D(u,v) [−], as shown in Equation (A5) [37]:

(A5) $$D\left(u,v\right)={\left(2E|X-Y|-E\left|X-X^{\prime }\right|-E\left|Y-Y^{\prime }\right|\right)}^{1/2},$$

where *X* ($X^{\prime }$) and *Y* ($Y^{\prime }$) are independent random variables whose probability distribution are *u* and *v*. The MMD has been applied to the PBM calibration in wet granulation [38] to capture the entire shape of distributions. While [38] used volume-based PSDs, the sum of the MMD of volume-based and number-based PSDs was used to capture the trends for fine particles. The model calibration procedure can be explained as an optimization problem:

(A6) $$\begin{array}{cc} \min_{\alpha ,\gamma ,\lambda } & D\left(m_{\mathrm{num}},s_{\mathrm{num}}\right)+D\left(m_{\mathrm{vol}},s_{\mathrm{vol}}\right) \end{array}$$

where the distributions $m_{\mathrm{num}}$, $s_{\mathrm{num}}$, $m_{\mathrm{vol}}$, and $s_{\mathrm{vol}}$ represent the number-based and volume-based PSDs for both measured (*m*) and simulated (*s*) data.

**Table A2:** Calibration summary of all the experiments with different APIs and process settings, where the objective function is the sum of MMD of number-based and volume-based PSDs.

API	GFR [m^3^/h]	MFR [g/min]	Objective Functions
Domperidone OG	2.2	5	0.135
	2.2	15	0.222
	5.6	10	0.257
	9.0	5	0.483
	9.0	15	0.204
Domperidone H1	2.2	5	0.086
	2.2	15	0.161
	5.6	10	0.071
	9.0	5	0.114
	9.0	15	0.082
Ketoconazole OG	2.2	5	0.497
	2.2	15	0.387
	5.6	10	0.616
	9.0	5	0.936
	9.0	15	0.728
Ketoconazole H1	2.2	5	0.366
	2.2	15	0.629
	5.6	10	0.521
	9.0	5	0.731
	9.0	15	0.339
Metformin H1	2.2	5	0.324
	2.2	15	4.407
	5.6	10	0.214
	9.0	5	0.117
	9.0	15	0.139
Metformin H2	2.2	5	0.314
	2.2	15	1.000
	5.6	10	0.188
	9.0	5	0.161
	9.0	15	0.438
Metformin H3	2.2	5	0.553
	2.2	15	0.481
	5.6	10	0.139
	9.0	5	0.319
	9.0	15	0.280
Indometacin	2.2	5	0.515
	2.2	15	0.365
	5.6	10	0.168
	9.0	5	0.584
	9.0	15	0.240
